# Risk factors for stoma outlet obstruction: systematic review and meta-analysis

**DOI:** 10.1007/s00423-025-03892-5

**Published:** 2025-10-29

**Authors:** Ali Toffaha, Ahmed Badr, Mahmood Al-Dhaheri, Ammar Aleter, Ejaz Latif, Mohamed Kurer, Ayman Ahmed, Noof Al Naimi, Issam Abu-Issa, Tausief Fatima, Amjad Parvaiz, Mohamed Abu Nada

**Affiliations:** 1 Colorectal Surgery Unit, General Surgery Department, Hamad Medical Corporation, Doha, Qatar; 2 College of medicine, Qatar University, QU health, Doha, Qatar; 3 General Surgery Department, Hamad Medical Corporation, Doha, Qatar; 4 School of Medicine, University of Science and Technology, Irbid, Jordan; 5 Department of colorectal surgery, Champalimaud Centre for Unknown, Lisbon, Portugal; 6 Faculty of Health Sciences, University of Portsmouth, Portsmouth, UK; 7 Department of Colorectal Surgery, Poole Hospital NHS Trust, Poole, UK

**Keywords:** Stoma outlet obstruction, Stoma-related obstruction, Stoma complications, Loop ileostomy, Causes, Risk factors

## Abstract

**Introduction:**

Stoma outlet obstruction (SOO) is a serious postoperative complication that can lead to significant morbidity, including prolonged hospitalization, increased healthcare costs, and reduced quality of life. This study, the first systematic review and meta-analysis on SOO, aims to identify and analyze key risk factors of SOO, calculate its pooled incidence, and systematically review its diagnostic features, clinical symptoms, imaging modalities, management strategies, prognosis, and associated outcomes.

**Methods:**

This systematic review and meta-analysis followed PRISMA 2020 guidelines and included 16 retrospective cohort studies, identified through a comprehensive search of multiple databases, with data on risk factors for SOO. The study analyzed four key variables reported by three or more studies, assessed study quality using the MASTER scale, and synthesized findings using the quality effects model to evaluate heterogeneity and publication bias.

**Results:**

This study included 16 retrospective cohort studies involving 2,228 patients, of whom 362 developed SOO. Increased rectus abdominis muscle thickness was found to significantly increase the risk of SOO (odds ratio [OR] 4.04, 95% confidence interval [CI] 2.36–6.93). High output stoma was another associated risk factor (OR 4.16, 95% CI 2.03–8.51). The type of ileostomy also played a critical role, with loop ileostomy showing a significantly higher risk of SOO compared to end ileostomy (OR 6.53, 95% CI 2.83–15.03). Although age was assessed as a potential risk factor, it did not show a statistically significant association with SOO (OR 1.69, 95% CI 0.44–6.54).

**Conclusion:**

This systematic review and meta-analysis identified significant risk factors for SOO, including increased rectus abdominis muscle thickness, high output stoma, loop ileostomy. We also reported other contributing factors, such as ileal pouch–anal anastomosis, shorter ileal pouch-to-ileostomy distance, oral inferior technique, smaller aperture size, higher BMI, and increased subcutaneous fat thickness. The findings emphasize the importance of tailored surgical techniques, such as stoma maturation using the oral superior technique, ensuring no twist at the mesentery, avoiding stoma limb angulation, creating the stoma slightly more proximally in cases of ileal pouch-anal anastomosis, and optimizing aperture size, along with vigilant postoperative care to reduce SOO incidence and improve patient outcomes.

**Supplementary Information:**

The online version contains supplementary material available at 10.1007/s00423-025-03892-5.

## Introduction

The construction of a stoma, such as a colostomy or ileostomy, is a common surgical procedure performed for various indications, including colorectal cancer, inflammatory bowel disease, diverticulitis, and traumatic injuries to the bowel. These procedures are essential for diverting fecal matter, allowing the distal bowel to heal, or as a permanent solution when the distal bowel is non-functional [1]. Stoma creation can be associated with complications, short term complication may include stoma retraction, necrosis, high output stoma (HOS) and stoma outlet obstruction (SOO); long term complications include stoma prolapse, stenosis, skin irritation and parastomal hernia [2].

SOO is a postoperative complication characterized by a blockage at the stoma limbs, which impedes the passage of intestinal contents [3]. This condition can lead to symptoms such as abdominal pain, distension, nausea, and vomiting, necessitating prompt medical intervention [3]. The terminology used to describe this condition varies widely, creating significant confusion. While the Common Terminology Criteria for Adverse Events (CTCAE) version 5.0 refers to it as “intestinal stoma obstruction [4],” other terms frequently used in the literature include “outlet obstruction [5, 6],” “stoma-related obstruction [7],” and “ileostomy obstruction [8],” among others. In this study, we have adopted the term “stoma outlet obstruction,” as it is the most commonly used [5, 6, 9–12].

The incidence of SOO varies across studies, with reported rates ranging from 5.4% to 25.8% [3, 9]. This variability can be attributed to differences in surgical techniques, patient populations, and definitions of obstruction. Consequences of SOO are significant and can include prolonged hospitalization, increased healthcare costs, and the potential need for additional surgical interventions. Moreover, SOO can adversely affect a patient’s quality of life, leading to nutritional deficiencies and delayed recovery [13]. Identifying risk factors associated with SOO is essential for developing preventive strategies and enhancing patient outcomes [14, 15]. A thorough understanding of these factors allows clinicians to customize surgical techniques and postoperative care to reduce the risk of this complication.

Given the substantial impact of SOO, its relatively high incidence, and the variability in reported risk factors across studies, this is the first systematic review and meta-analysis to be conducted to address these gaps. The primary objectives were to identify and analyze the key causes and risk factors associated with SOO and to estimate its pooled incidence. Secondarily, the study systematically reviewed the diagnostic features, clinical symptoms, imaging methods, management strategies, prognosis, and associated outcomes. By synthesizing data from multiple studies, this analysis provides a robust understanding of the factors contributing to SOO, offering insights to help colorectal surgeons optimize surgical techniques, implement targeted interventions for high-risk patients, and ultimately improve patient outcomes while reducing the burden of SOO.

## Methods

### Study design and protocol registration

This is a systematic review and meta-analysis that followed the Preferred Reporting Items for Systematic Reviews and Meta-Analyses (PRISMA) 2020 guidelines. The PRISMA checklist can be found in the Supplementary Table S1. The protocol for this review was registered on the International Prospective Register of Systematic Reviews (PROSPERO) database with registration ID (CRD42025632002).

### Search strategy and data sources

The search strategy for this systematic review and meta-analysis was generated using the keywords (“stoma outlet obstruction,” “stomal obstruction,” “stoma-related obstruction,” “stoma outlet,” “stoma complications,” “ileostomy,” “loop ileostomy,” “causes,” “risk factors”) in multiple electronic databases via a polyglot translator: PubMed, Embase, Scopus, Cochrane Library, and CINAHL Ultimate. During the initial search, we did not impose any restrictions on study type, language, or publication date. The full search strategy for each database is presented in the supplementary material. The last search was conducted on 29th of November 2024. The resulting articles were firstly imported into Endnote (Clarivate, London, UK) to check for any duplicates and then uploaded to the Rayyan Systematic Review Management platform (Rayyan Systems, Inc., Cambridge, MA, USA) for the screening process.

### Study selection and screening

Preliminary screening of the retrieved articles was done independently by two investigators (A.T. and A.B.) using titles and abstracts through Rayyan platform. As the screening process was blinded and done manually, any discrepancies between the two investigators were then resolved through consensus. Then, the study records that were identified from the titles and abstracts underwent full-text screening by (A.T. and A.B.).

### Eligibility criteria

Articles reporting patients, post colorectal surgery with diverting loop ileostomy, who developed SOO with a control group of individuals who did not develop obstruction were reviewed. The PECO question of our study can be highlighted as the following: P (patients with diverting ileostomies), E (presence of risk factors associated with SOO), C (Patients with ileostomies who did not develop SOO), O (SOO development). For these articles to be included, there should be a comparison of the different factors associated with the development of SOO between the two groups using a multivariate analysis. Reported factors within each study must be included in the study’s multivariate analysis to be eligible for the inclusion within our study. Around two thousand and seven hundred articles were screened, with only sixteen studies succeeded to meet our eligibility criteria. Exclusion criteria were case reports/series, studies which did not conduct a multivariate analysis for the reported factors, studies on patients with colostomy only, abstracts, editorials, and letters to editor. We also identified other significant risk factors for SOO during the literature review that were not included in our study’s analysis due to the eligibility criteria but were reported separately.

### Data extraction

SOO was described using varied terminology across the included studies. Terms such as outlet obstruction, stoma outlet obstruction, bowel obstruction at the stoma opening, narrowing of the stoma, tightening of the stoma outlet, failure to discharge from the stoma, stenosis at the stoma site, stoma-related obstruction, stoma-associated small bowel obstruction, ileostomy dysfunction, and intestinal stoma obstruction were all used to refer to SOO. There are certain characteristics that were extracted from every study: authors, year of publication, study design, study groups, country in which the study was performed, total number of patients included, and the number of patients who developed obstruction. Furthermore, we extracted the factors that met the eligibility criteria from each study. Although many risk factors are reported in the included studies, twenty-seven variables met our eligibility criteria. Due to the low number of included studies, only four variables were reported by three or more articles and were added to our data analysis. Data were compiled in an online Excel spreadsheet and was accessible to all contributing authors.

### Outcomes

The primary objective of our study was to identify and analyze the key causes and risk factors associated with SOO. Factors reported in at least three studies were evaluated to determine the most significant contributors to SOO, providing valuable insights to colorectal surgeons. These findings aim to reduce the incidence and burden of SOO through optimizing surgical techniques and improve postoperative care for high-risk patients. Furthermore, we calculated the pooled incidence of SOO across the included studies to provide a comprehensive estimate. As a secondary objective, we systematically reviewed and analyzed the diagnostic features, clinical symptoms, imaging modalities, timing, and management strategies for SOO reported in the selected studies. Additionally, we examined the prognosis, prolonged hospitalization, and reoperation rates among patients who developed SOO.

### Quality assessment of the included studies

The quality of the included studies was evaluated independently by two investigators (A.T. and A.B.) using the Methodological Standard for Epidemiological Research (MASTER) scale, which consists of 36 safeguards categorized into seven methodological standards. Discrepancies between investigators were resolved through discussion. The seven domains of the MASTER scale are: equal recruitment (safeguards 1–4), equal retention (5–9), equal ascertainment (10–16), equal implementation (17–22), equal prognosis (23–28), sufficient analysis (29–31), and temporal precedence (32–36).

### Data analysis

The quality effects model, which adjusts for bias, was used to synthesize the outcome estimates. This model applies inverse variance heterogeneity meta-analysis synthesis, assuming all studies estimate a common effect. It adjusts for variability arising from differences in methodological quality among the different studies by redistributing study weights based on quality rankings. Results of the quality effects model, outcome estimates and their pooled value, were displayed using forest plots. Heterogeneity was assessed using the I² statistic and the Cochrane Q test, with statistically significant heterogeneity defined as *P* < 0.05 for the Cochrane Q test or I² >50%. Publication bias was evaluated using Doi plots, the Luis Furuya-Kanamori (LFK) index, and funnel plots. Statistical analyses were done using Stata 18 (StataCorp, College Station, TX, USA).

### Ethical approval

This review utilized secondary data from peer-reviewed published studies, eliminating the need for ethical approval.

## Results

### Study selection

**Fig. 1:**
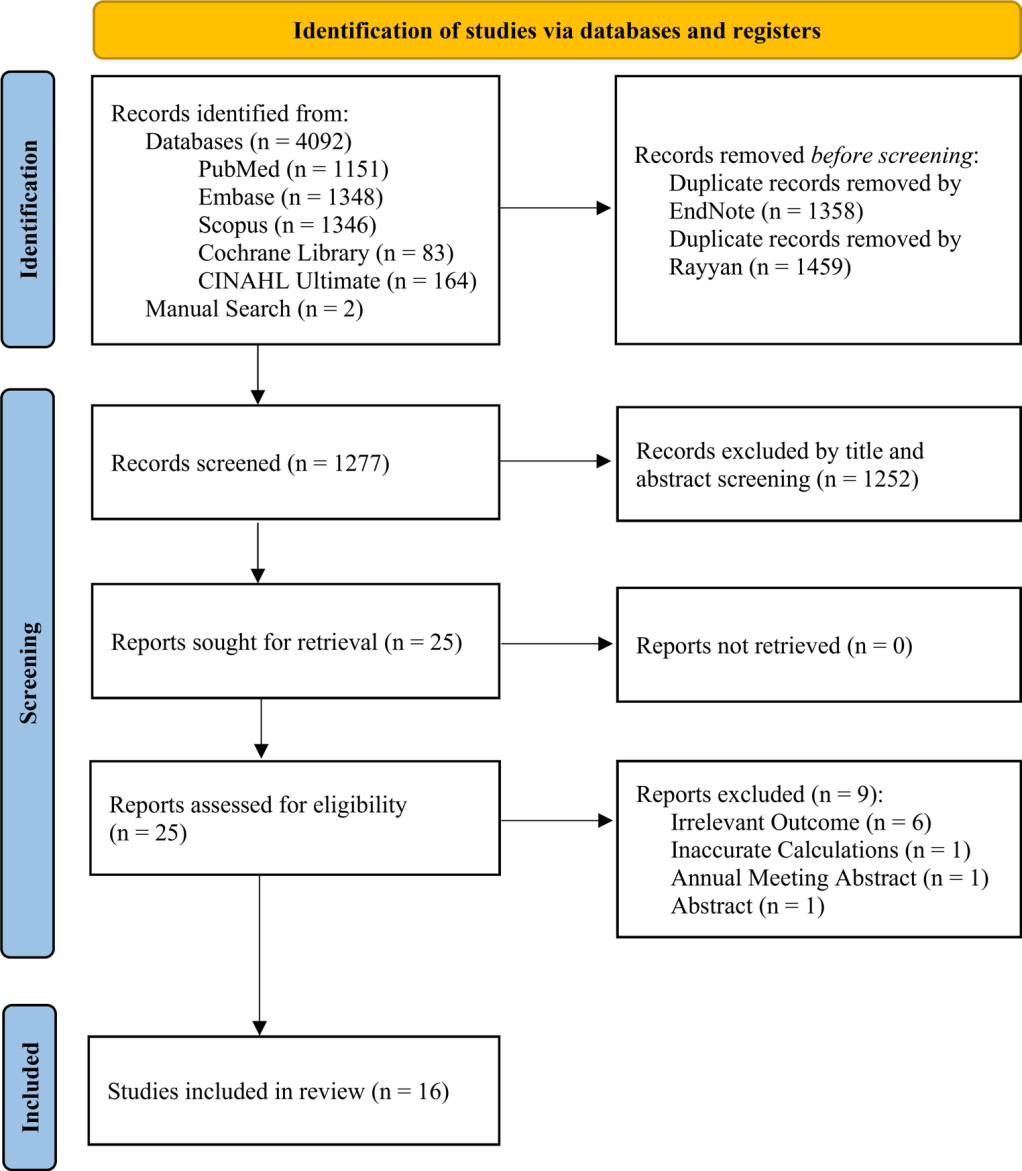
PRISMA flowchart shows the selection process in our systematic review. PRISMA, preferred reporting items for systematic reviews and meta-analyses

The search process and study selection followed the PRISMA flowchart (Fig. 1). A total of 4092 records were identified from the electronic searches of the specified databases. An additional two studies were identified through manual search. After removing 1358 duplicate records using EndNote, the remaining 2736 records were transferred to Rayyan platform. After removing extra 1459 duplicates through Rayyan, 1277 distinctive articles remained. Title and abstract screening led to the exclusion of 1252 records. Nine of the remaining 25 studies were removed after doing full-text screening for the reasons mentioned in the PRISMA chart (Fig. 1). Full-text screening was conducted by two independent investigators (A.T. and A.B.). Details regarding the excluded articles at the full-text screening process are available in Supplementary Table S2. Finally, sixteen articles met the selection criteria and were included in this systematic review.

### Characteristics of included studies

This systematic review included 16 retrospective cohort studies, encompassing a total of 2,228 patients, of whom 362 experienced SOO. Fifteen studies were conducted in Japan, with one study (Caprino et al.) performed in Italy [16]. The studies compared various factors between patients with SOO and those without, aiming to identify significant risk factors for obstruction. The extracted characteristics include patient demographics, surgical details, and postoperative outcomes. Key variables examined in the studies include patient age, sex, stoma type (loop vs. end ileostomy), rectus abdominis (RA) muscle thickness, high output stoma (HOS), and other preoperative and intraoperative factors. Among these, only a few variables were consistently reported across multiple studies, allowing for meta-analysis. Further details can be found in Table 1.

The study populations among the included studies varied in size, with sample sizes ranging from 68 to 261 patients per study. Several factors, such as RA muscle thickness and the presence of HOS, were found to be strongly associated with an increased risk of SOO. Additionally, differences in stoma positioning and surgical techniques were evaluated to determine their role in the development of obstruction. Overall, these studies provided comprehensive data on patient and procedural variables, which were analyzed to identify high-risk groups and inform clinical practices for reducing the incidence of SOO.

### Quality assessment

**Fig. 2:**
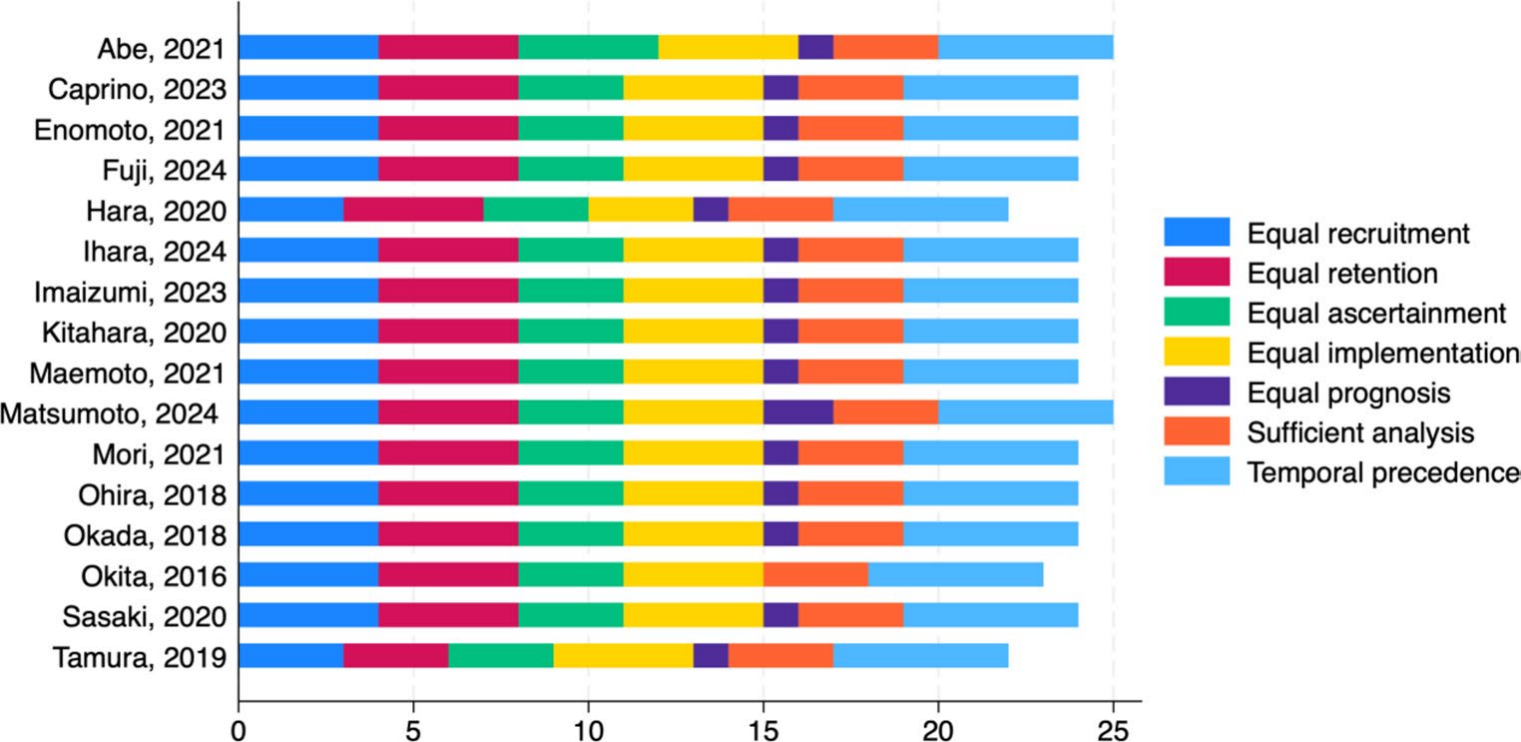
Quality scores for the included studies

Across the included studies, the number of safeguards fulfilled ranged from 23 to 25 (mean 24), indicating a generally moderate-to-high methodological quality. A higher score indicated better quality, as it reflected a greater number of safeguards against systematic error. As illustrated in Fig. 2, most studies scored consistently well in equal recruitment, temporal precedence, and sufficient analysis. In contrast, safeguards related to equal ascertainment and equal prognosis were less frequently achieved, reflecting inherent limitations of retrospective study designs. The narrow range of scores suggests not only moderate-to-high overall quality but also methodological consistency across studies, thereby minimizing heterogeneity arising from study quality. Detailed assessments for each study are provided in Supplementary Table S3.

### Primary outcome

From the included studies, four remarkable risk factors were analyzed as there were three or more studies reporting the same risk factor which fulfills the eligibility criteria in our meta-analysis. These four risk factors were: RA muscle thickness, HOS, type of ileostomy, and age. We also calculated the pooled incidence of SOO which was around 16.25%. This reflects the average rate of SOO occurrence across all studies included in the analysis.

**Fig. 3:**
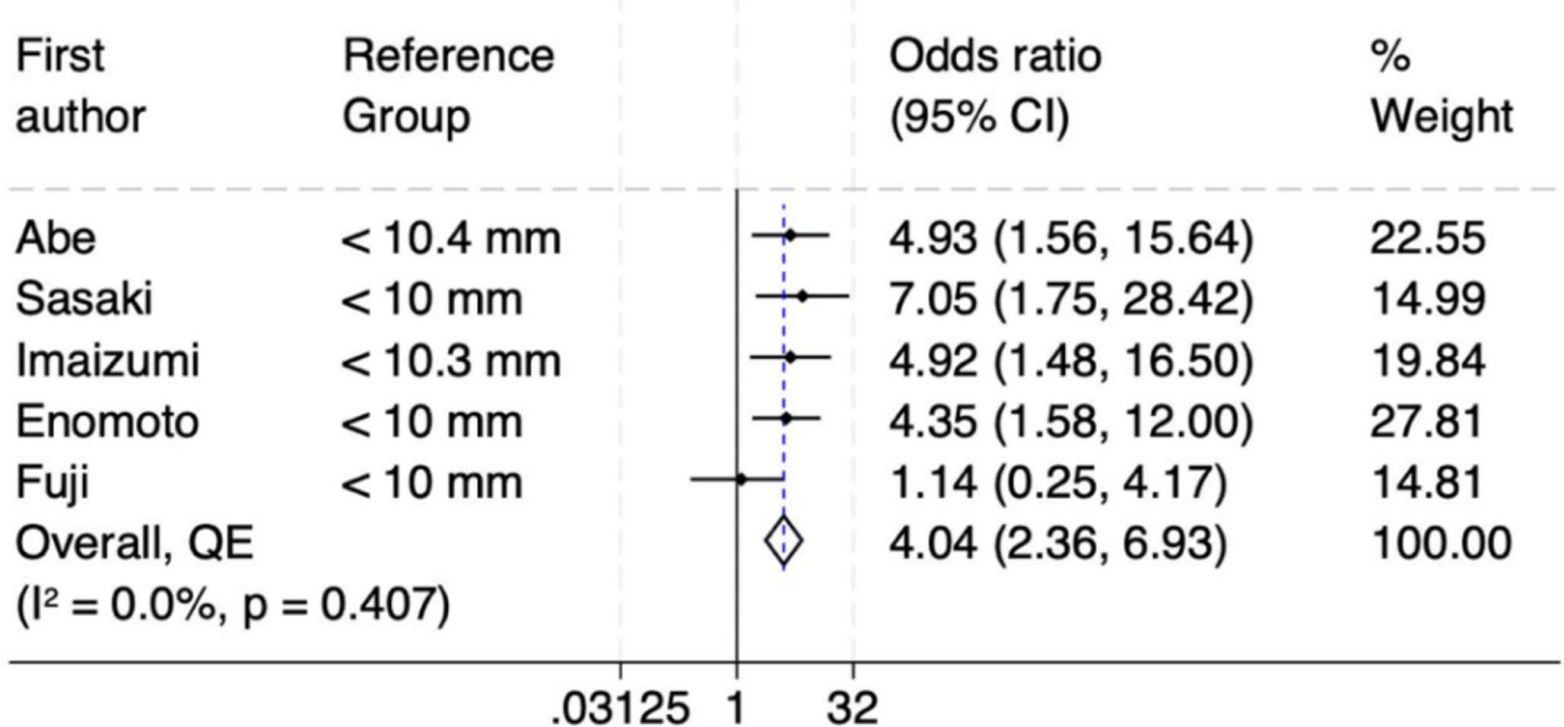
Forest plot of rectus abdominis muscle thickness as a risk factor for SOO

Firstly, RA muscle thickness was reported by five studies and found to be a significant risk factor for developing SOO [9, 10, 12, 17, 18]. The forest plot (Fig. 3) reveals a statistically significant pooled odds ratio of 4.04 (95% CI: 2.36–6.93), indicating that individuals with greater RA muscle thickness (> 10.4 mm) have over four times the odds of developing SOO compared to those with thinner muscles. The overall result is statistically significant since the CI does not include the null value 1. The lack of heterogeneity among the studies (I² = 0.0%, *p* = 0.407) reinforces the reliability of this association across the included studies. The funnel plot demonstrates some asymmetry, with a noticeable absence of smaller studies with negative or insignificant findings on the left side of the plot, hinting at potential publication bias or systematic differences (Supplementary Figure S1). However, the LFK index of −0.89 from the DOI plot suggests no significant asymmetry, indicating minimal risk of publication bias (Supplementary Figure S2). Despite this, the findings remain robust due to the strong pooled effect size, absence of heterogeneity, and minimal asymmetry indicated by the LFK index. Kuwahara et al. also reported that patients with a thicker RA muscle were at a higher risk of developing SOO [19]. They suggested that SOO was linked to the medial inclination of the abdominal incision wall, which creates increased tension due to the unfavorable angles at the stoma site.

**Table 1:** Characteristics of included studies

First author	Study Design	Country	TP	OP	Age	BMI	male/female	primary diagnosis	surgical procedure performed	indication for ileostomy	incidence of SOO
**Caprino**,** 2023** [16]	Retrospective cohort	Italy	75	20	44 ± 15.6	21.9 ± 3.8	46/29	UC	RPC and IPAA with diverting ileostomy	part of the surgery	26.70%
**Matsumoto**,** 2024** [20]	Retrospective cohort	Japan	188	28	63.4 ± 11.6	N/A	136/52	CRC	Primary resection of rectal cancer and Loop ileostomy	Initial decompression of stenosis at the primary site, and prevent anastomotic leakage	14.90%
**Mori**,** 2021** [21]	Retrospective cohort	Japan	68	18	49.0 ± 16.5	20.8 ± 4.3	45/23	UC	TPC and IPAA with diverting ileostomy	avoid the risk of anastomotic leakage following IPAA	26.50%
**Tamura**,** 2019** [22]	Retrospective cohort	Japan	230	16	65	22.2	164/66	RC	LAR with defunctioning loop ileostomy	prevent severe anastomotic leakage	7.00%
**Hara**,** 2020** [14]	Retrospective cohort	Japan	103	19	66	22.9	82/21	malignant tumor, IBD, and colon perforation	LAR, ISR, and TPC	reduce the risk of anastomotic leakage	18.40%
**Maemoto**,** 2021** [3]	Retrospective cohort	Japan	155	12	63 ± 13	23.1 ± 4.4	104/51	RC, UC, and FAP	LAR, ISR, TPC, and IPAA	reduce the risk of anastomotic leakage, part of the surgery	7.70%
**Okita**,** 2016** [7]	Retrospective cohort	Japan	205	53	37 ± 15	20.0 ± 3.6	115/90	UC	RPC and IPAA with diverting ileostomy	part of the surgery	25.80%
**Kitahara**,** 2020** [23]	Retrospective cohort	Japan	148	25	48	20.1	95/53	UC	RPC with IPAA, Colectomy with ileorectal anastomosis, Colectomy with end ileostomy, Proctocolectomy with permanent end ileostomy, Proctectomy with end ileostomy	Standard practice for staged procedures (two or three-stage) to protect anastomoses, particularly in patients on high-dose glucocorticoids/immunosuppressants or who are undernourished	16.90%
**Ihara**,** 2024** [11]	Retrospective cohort	Japan	68	11	46.0 ± 18.6	19.9 ± 3.5	38/30	UC	RPC with diverting ileostomy	protect the ileoanal anastomosis	16.20%
**Okada**,** 2018** [24]	Retrospective cohort	Japan	225	26	N/A	N/A	N/A	RC and UC	IPAA and LAR	prevent severe anastomotic leakage	11.50%
**Abe**,** 2021** [17]	Retrospective cohort	Japan	125	20	60	22.2	78/47	RC and GIST/NET	LAR, sLAR, ISR, and TPC	prevent postoperative anastomotic leakage	16.00%
**Sasaki**,** 2020** [18]	Retrospective cohort	Japan	261	14	59	N/A	164/97	RA	Laparoscopic primary resection + Double stapling technique anastomosis or Hand-sewn colo-anal anastomosis	neoadjuvant therapy, very low anastomoses, technical difficulties (poor bowel preparation, anastomotic tension, positive air leak test, and incomplete anastomotic ring)	5.40%
**Imaizumi**,** 2023** [12]	Retrospective cohort	Japan	92	24	62	21.4	57/35	RC and NET	Primary resection of rectal cancer and defunctioning ileostomy	prevent symptomatic anastomotic leakage	26%
**Ohira**,** 2018** [25]	Retrospective cohort	Japan	107	18	61	21	60/47	UC and RC	TPC and LAR	prevent or manage anastomotic leakage	16.80%
**Enomoto**,** 2021** [9]	Retrospective cohort	Japan	100	28	60.5	22.1	73/27	RC	Anterior resection and LAR	very low anastomoses, technical difficulties (poor bowel preparation, anastomotic tension, positive air leak test, and incomplete anastomotic ring)	28.00%
**Fujii**,** 2024** [10]	Retrospective cohort	Japan	78	12	69	22.3	59/19	RC, UC, FAP and GIST/NET	sLAR and TPC	very low anastomoses and prevent severe anastomotic leakage	15.40%

TP: Total population; OP: Obstruction Population; SOO: Stoma Outlet Obstruction; UC: Ulcerative Colitis; RPC: Restorative proctocolectomy; IPAA: Ileal Pouch–Anal Anastomosis; CRC: Colorectal Cancer; TPC: Total proctocolectomy; RC: Rectal Cancer; LAR: Low Anterior Resection; IBD: Inflammatory Bowel Disease; ISR: Intersphincteric Resection; FAP: Familial Adenomatous Polyposis; RPC: Restorative proctocolectomy; GIST: Gastrointestinal Stromal Tumors; NET: Neuroendocrine Tumors; sLAR: Super Low Anterior Resection; RA: Rectal Adenocarcinoma

**Fig. 4:**
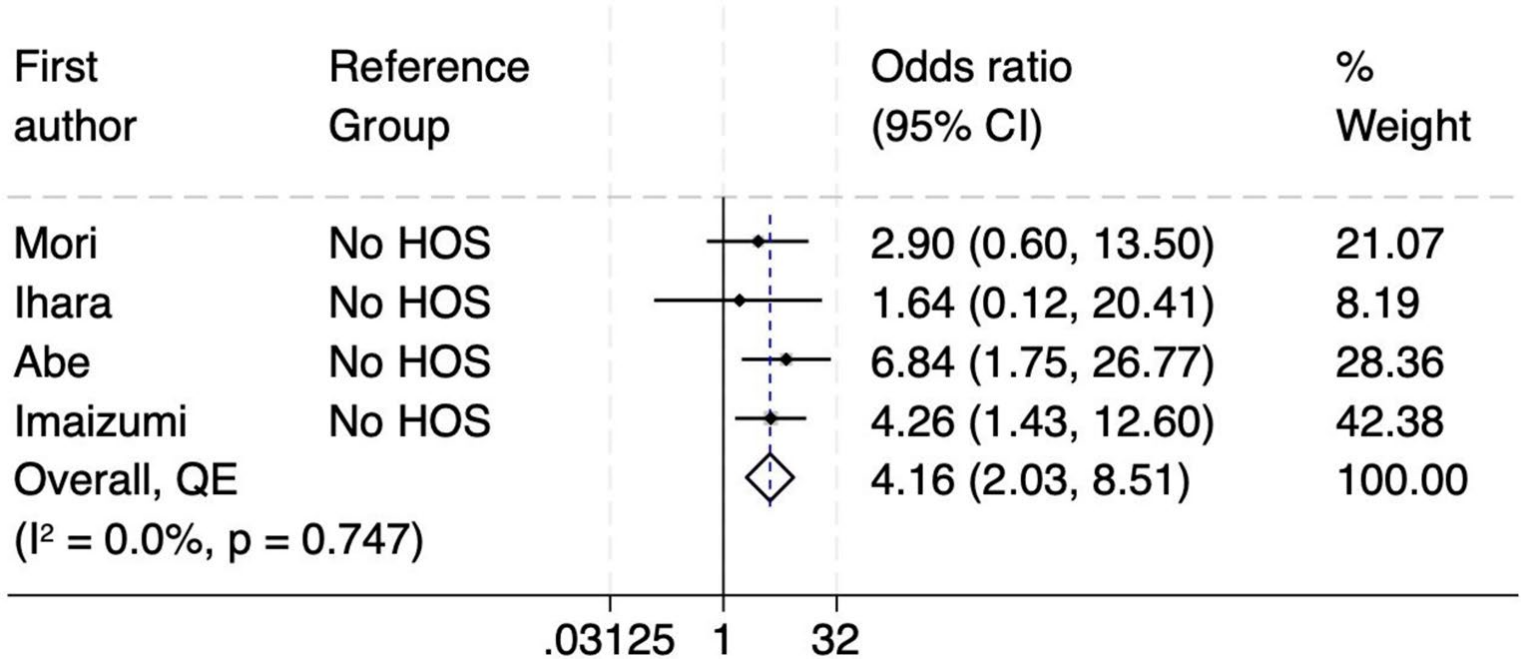
Forest plot of HOS as a risk factor for SOO

Secondly, HOS is clinically defined as a stoma output more than 1500–2000 mL/24h [11, 12, 17, 21]. This can lead to significant fluid and electrolyte imbalances, requiring prompt medical management. The forest plot in Fig. 4 demonstrates the association between HOS and the risk of obstruction across four studies [11, 12, 17, 21]. The pooled odds ratio is 4.16 (95% CI: 2.03–8.51), suggesting that individuals with a HOS are over four times more likely to experience obstruction compared to those without. The heterogeneity among the studies is negligible, as indicated by an I² value of 0.0% and a p-value of 0.747, suggesting consistency in findings across the included studies. The funnel plot and DOI plot further evaluate potential publication bias (Supplementary Figures S3 & S4). The LFK index is −2.06, indicating major asymmetry. This, along with the visual asymmetry observed in the funnel plot, suggests a moderate to high risk of publication bias, which could affect the robustness of the pooled estimate.

**Fig. 5:**
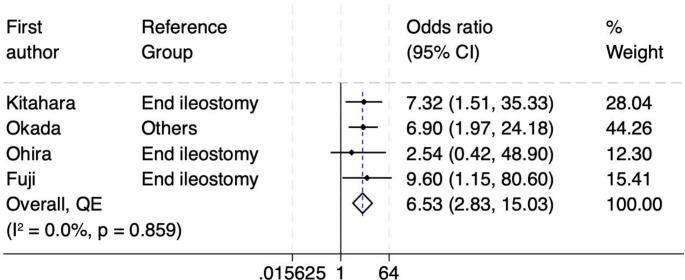
Forest plot of ileostomy type (loop vs. end) as a risk factor for SOO

Thirdly, the type of ileostomy is being assessed as an independent risk factor for SOO [10, 23–25]. Figure 5 shows the forest plot that evaluates the association between the type of ileostomy (loop vs. end) and the risk of SOO. The pooled odds ratio is 6.53 (95% CI: 2.83–15.03). This indicates that patients with a loop ileostomy have a significantly higher risk, over six times greater, of developing SOO compared to those with an end ileostomy. Heterogeneity was assessed using the I² statistic and the Cochrane Q test, with an I² value of 0.0% and a p-value of 0.859. These results indicate no significant heterogeneity, meaning the findings are consistent across studies. The funnel plot and DOI were used to assess publication bias (Supplementary Figures S5 & S6). The LFK index is −0.84, indicating no asymmetry, which is confirmed by the symmetrical funnel plot. This suggests a low risk of publication bias, supporting the reliability of the pooled odds ratio.

**Fig. 6:**
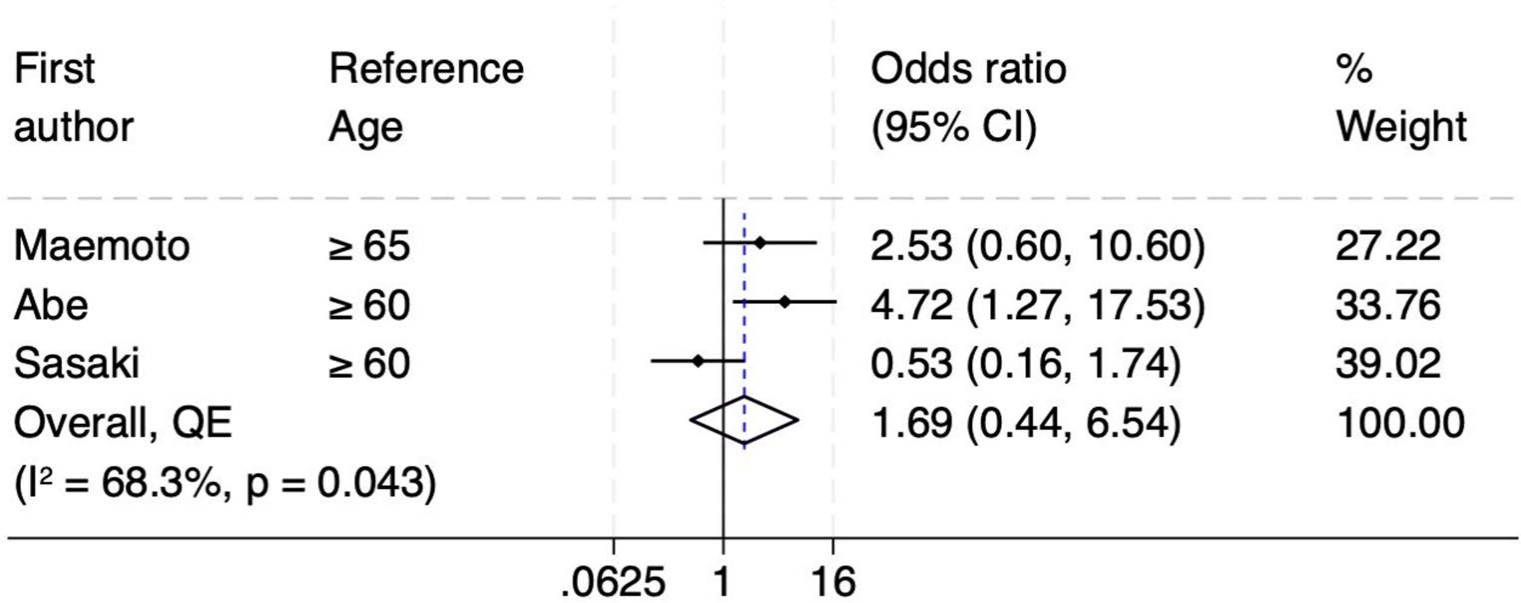
Forest plot for age as risk factor for SOO

Finally, age was not found to be an independent risk factor for developing SOO. As can be seen in Fig. 6, among the individual studies, Abe et al. shows a statistically significant association, while Maemoto et al. and Sasaki et al. do not, as their confidence intervals include the null value [3, 17, 18]. The pooled odds ratio is 1.69 (95% CI: 0.44–6.54), indicating no statistically significant overall association. Moderate heterogeneity as indicated by an I² value of 68.3% and a significant Cochrane Q test p-value of 0.043, suggests variability in study populations or methodologies, which could influence the combined results. The funnel plot shows a roughly symmetrical distribution of studies around the pooled effect size, with no significant visual evidence of publication bias. Additionally, the LFK index of −0.48 supports the absence of asymmetry, further confirming that publication bias is unlikely to have influenced the meta-analysis results (Supplementary Figures S7 & S8). Overall, while one study (Abe et al.) demonstrates a significant association between age and SOO, the pooled evidence does not support age as a definitive risk factor.

### Other risk factors

There was other risk factors associated with SOO but were not included in the analysis of this study. These factors were only reported with no multivariate analysis done which hinders adding them to our meta-analysis. Firstly, Okada et al. reported that patients who have ileal pouch–anal anastomosis (IPAA) have higher risk of developing SOO reaching up to 47% in IPAA patients [24], compared to risk of 8.7% to 23% in cases of rectal resection [26–32]. Secondly, the distance between the ileal pouch and the ileostomy is a significant risk factor; Mizushima et al. reported that the incidence of SOO was notably greater among patients with an ileal pouch-to-ileostomy distance of less than 30 cm, as the tension increases [32]. In addition, some surgical techniques while constructing the stoma increases the risk of SOO; Takehara et al. showed that the oral inferior (OI) method of constructing the stoma causes the stoma to twist and leads to higher rates of SOO compared with the oral superior (OS) technique [33]. Also, the aperture size of the abdominal wall plays an important role; It was reported by Matsumoto et al. that a smaller stoma diameter at the rectus abdominis level on CT was significantly associated with higher risk of SOO [20]. Finally, BMI and subcutaneous fat thickness were also reported by Tamura et al. as independent factors for SOO [22]; Subcutaneous fat at the stomal site > 20 mm and BMI > 22.2 were found to increase the risk of SOO.

### Secondary outcome

SOO is diagnosed primarily through clinical symptoms and imaging findings. Common symptoms reported across the included studies include abdominal pain, distension, nausea, vomiting, and reduced or absent fecal discharge through the stoma [3, 20]. In some cases, persistent stoma output, albeit thinner than usual, complicates early detection as reported by Hara et al. [14]. Computed tomography (CT) is the most frequently utilized diagnostic modality, identifying features such as dilated small bowel loops proximal to the obstruction or caliber changes at the stoma site [11]. Other imaging methods, such as contrast studies through the stoma, are used to exclude other causes of obstruction, including adhesions, paralytic ileus, or internal hernias, aiding in precise diagnosis [23]. Across several of the included studies, CT emerged as the most consistent and reliable diagnostic tool for SOO. Fujii et al. and Ohira et al. both demonstrated that CT could clearly localize the obstruction to the stoma site, while Enomoto et al. highlighted its value in distinguishing SOO from paralytic ileus in the immediate postoperative setting [5, 9, 25]. Taken together, these findings support CT as the gold-standard modality for confirming SOO and guiding appropriate intervention. The accurate identification of SOO relies on combining clinical assessments with imaging findings, meeting at least two key criteria. Firstly, the presence of initial symptoms of bowel obstruction or ileus, and secondly, either relief of obstruction following the insertion of a trans-stomal decompression catheter or CT imaging evidence showing a transition point at the level of the abdominal wall without signs of other obstructions [33]. In addition, the onset of SOO typically occurs within the first two weeks following surgery, with most cases presenting between postoperative days 2 and 7 [17, 22]. Differentiating SOO from other causes of bowel obstruction, such as adhesive disease or internal hernia, is particularly challenging during the early postoperative period [10]. These findings highlight the need for vigilant monitoring during this critical window to ensure timely diagnosis and intervention, reducing the potential for prolonged symptoms or complications.

The management of SOO varies depending on symptom severity and the underlying cause. Conservative measures, including bowel rest, intravenous fluid therapy, and decompression using a tube inserted through the stoma, have proven effective in most cases as reported by many studies [7, 18]. Imaizumi et al. reported that early stoma closure is often recommended for patients with recurrent or refractory symptoms [12]. However, in severe cases or when conservative management fails, surgical intervention, such as stoma reconstruction or laparotomy, may be required as suggested by Ohira et al. [25]. Also, the analyzed studies emphasize the role of preventive strategies, such as optimizing stoma height and avoiding excessive tension during stoma construction, to reduce the risk of SOO. When managed promptly, most patients experience symptom resolution and favorable outcomes, underscoring the importance of early diagnosis and tailored interventions [21]. Prolonged hospitalization is a frequent consequence of SOO, significantly impacting patient recovery and healthcare resources. Matsumoto et al. reported that the mean hospital stay for SOO patients was 29.3 days, compared to 17.5 days for those without obstruction, representing a substantial delay in recovery​ [20]. Similarly, Imaizumi et al. found a median hospitalization duration of 31 days for SOO patients, compared to 18 days in non-SOO patients [12]. Okita et al. observed that over half of the patients with SOO (58%) required extended hospital stays beyond the typical postoperative period due to persistent symptoms such as abdominal distension and nausea [7].

The need for reoperation is another possible critical consequence of SOO, as conservative treatments may fail in severe or recurrent cases. Maemoto et al. documented that 67% of SOO patients required surgical intervention, such as early stoma closure or revision, to resolve the obstruction [3]. Ohira et al. reported that 44% of patients with SOO underwent reoperation, with procedures including stoma reconstruction and laparotomy commonly performed [25]. Additionally, Sasaki et al. highlighted that patients undergoing reoperations faced a 25% risk of postoperative complications, such as surgical site infections and adhesion-related issues​ [18]. The type of surgical procedure and the timing of reoperation play a significant role in determining patient outcomes. Fujii et al. reported that patients who underwent surgical correction within two weeks of symptom onset had an 85% rate of symptom resolution within four weeks [10]. In contrast, Tamura et al. noted that delays in addressing SOO resulted in prolonged recovery, with hospital stays exceeding six weeks in 30% of cases [22]. Early intervention appears to mitigate the duration and severity of SOO-related complications but delays often necessitate more complex surgical procedures and result in longer recovery times.

Several studies also explored specific factors contributing to prolonged hospitalization and reoperation. Okada et al. highlighted that patients with IPAA were more likely to require surgical correction due to the development of SOO compared to those with diverting loop ileostomy [24]. Mori et al. found that a shorter distance between the stoma and the ileal pouch was associated with increased rates of reoperation, suggesting that anatomical factors play a critical role in the development and progression of SOO [21]. In terms of prognosis, studies generally reported favorable outcomes following timely intervention. Imaizumi et al. observed that 92% of SOO patients who received early decompression or surgical correction experienced symptom resolution without long-term complications [12]. As noted by Hara et al., delayed treatment was associated with worse outcomes, including increased rates of recurrence and reduced quality of life [14]. These findings emphasize the importance of early diagnosis and prompt management to minimize the impact of SOO on patient recovery and overall prognosis.

## Discussion

### Key findings

This study identified many significant risk factors for SOO, including increased RA muscle thickness, high output stoma (HOS), and loop ileostomy. Patients with thicker RA muscle at stoma site were found to have approximately four times higher odds of developing SOO compared to those with thinner muscle thickness. This finding could be explained by the increased length of the stoma limbs within the RA muscle, which are more likely to be compressed by the surrounding muscle tissue. This compression can result in the intestinal pressure being exceeded by the pressure exerted by the RA muscle, ultimately hindering the excretion of stoma output. Similarly, as the thickness of the RA muscle increases, the angle between the stoma tunnel and the abdominal cavity becomes sharper, contributing to further risk of obstruction [33]. Moreover, patients with HOS were over four times more likely to experience SOO. The relationship between SOO and HOS can be attributed to a shared risk factor, as explained by Hara et al., namely surgical site infection (SSI) [14]. SSI can result in intestinal edema and a prolonged reduction in intestinal absorption, both of which may play a role in the development of HOS and SOO, contributing to their interconnected pathology. Consequently, patients with organ/space SSI should be managed with careful consideration of the potential risk of developing HOS and SOO. Another explanation is that the rapid increase in stoma output observed in HOS leads to mucosal edema, which subsequently causes a relative obstruction [12, 17]. However, this relationship should be further studied and proven, as HOS could be just a surrogate for intermittent obstruction and overflow, before the full-blown obstruction manifest.

The type of stoma was another significant factor, with patients undergoing loop ileostomy having more than six times higher odds of developing SOO compared to those with diverting end ileostomy (DEI). This finding highlights the mechanical nature of SOO, which primarily occurs at the level of the abdominal wall. In the case of DEI, the reduced volume of intestinal and mesenteric segments passing through the stoma aperture may lower the risk of SOO, underscoring the potential protective effect of this configuration [10]. Colostomies were found generally to have much lower risk of SOO by many studies, owing to the higher intraluminal pressure in the colon compared to small bowel, which usually overcomes the outlet constriction [3, 17, 18]. Looking at patient’s age, some individual studies have suggested that SOO may be more prevalent in younger age groups, potentially due to their thicker and stronger abdominal wall muscles [3, 17, 18]. In our meta-analysis, and when evaluated through multivariate and pooled analyses, age itself did not emerge as an independent risk factor, indicating that other factors, such as muscle thickness, may have a more direct influence on the development of SOO.

In addition to the three primary risk factors identified in this meta-analysis, our systematic review highlights several additional potential factors that warrant further investigation through larger-scale studies. Among these, IPAA was associated with a higher risk of SOO compared to the construction of diverting loop ileostomy for rectal resection, particularly when the distance between the stoma and the pouch was shorter [24, 31, 32]. This increased risk may stem from the anatomical and mechanical differences during the stoma construction between the two procedures. In IPAA, the stoma is created more proximally in the small bowel, and the distal ileal fixation in the pelvis creates tension that hinders spontaneous resolution of angulations at the stoma limbs [3, 24, 32]. In contrast, rectal resection does not involve such distal ileal fixation, leaving the ileocecal area intact. Ihara et al. further theorized that the removal of the whole colon in IPAA cases increases the available space, allowing stoma limbs to twist more easily [11].

Both Ihara et al. and Ohira et al. identified laparoscopic surgery as a significant risk factor for SOO [11, 25]. Ihara et al. reported that patients undergoing laparoscopic proctocolectomy were over seven times more likely to develop SOO compared to those who had open surgery [11]. They hypothesized that this could be due to the increased mobility of the small intestine postoperatively, leading to a greater tendency for twisting or angulation at the stoma outlet, which can impede intestinal flow. Similarly, Ohira et al. found that patients who underwent laparoscopic surgery had a nearly threefold increased risk of developing SOO, likely due to the increased movable area of the small intestine, which increases the risk of torsion and obstruction [25]. Moreover, SOO has become more prevalent with the rise of minimally invasive surgical techniques. One proposed explanation is that during laparoscopic surgery, the abdominal cavity is inflated, making the bowel loop chosen for stoma appear straighter and properly positioned. However, when the abdomen is deflated at the end of surgery, the bowel may angulate and become trans-oblique to the abdominal wall, leading to a functional obstruction at the stoma outlet.

Rotation during stoma creation has emerged as a notable potential risk factor for SOO. The oral inferior (OI) technique, in which the oral side of the stoma is positioned at the 6 o’clock position, has historically been favored for its ability to prevent stool inflow into the anal side and facilitating self-care [33]. However, this technique is associated with a higher risk of SOO. In the OI configuration, the oral-side intestine tends to descend into the pelvis when the patient is upright, leading to stool pooling in the oral side, creating a gravitational pull that drags the proximal limb downward. This increases the likelihood of the proximal limb to bend at the transition point between the stoma tunnel and the abdominal cavity, which may result in obstruction. In contrast, the oral superior (OS) technique, where the oral side of the stoma is positioned at the 12 o’clock position, has been associated with a lower risk of SOO. This configuration reduces gravitational pull on the proximal limb and shifts compression to the anal limb if the oral side is drawn downward. The OS arrangement allows the proximal limb to utilize the stoma tunnel size more effectively, minimizing the risk of narrowing or obstruction. These findings suggest that the OS technique provides a biomechanical advantage in reducing SOO [33].

Aperture size, BMI, and subcutaneous fat thickness have been identified as additional potential risk factors for SOO, all sharing a common mechanism of relative mechanical constriction at the stoma outlet. Among these, aperture size stands out as the most modifiable factor, allowing surgeons to directly address the risk of SOO during stoma creation. Matsumoto et al. demonstrated that larger stoma incisions in the fascia are associated with a reduced risk of SOO [20]. However, this benefit comes with an increased incidence of parastomal hernia, which tends to rise in parallel with the size of the incision. Their findings suggest an optimal incision size of approximately 40 mm to balance the risks of SOO and hernia [20]. Given that most diverting loop ileostomies are temporary, the risk of parastomal hernia is often less concerning in such cases. Enlarging the aperture size to prevent SOO may be a more practical approach, particularly since SOO can significantly prolong hospital stays and delay crucial treatments such as adjuvant chemotherapy [10]. These findings collectively provide a strong foundation for evidence-based improvements in surgical planning, stoma construction, and postoperative management to mitigate the risk of SOO.

In terms of presentation and diagnosis, mucosal edema typically becomes most prominent on the third or fourth postoperative day, aligning with the timing of SOO onset. Edema usually subsides around the seventh postoperative day, and if the decompression tube is removed after this point, the stoma can often remain tube-free without a recurrence of SOO. This observation supports the role of mucosal edema as a contributing factor in the development of SOO [9]. It is also important to recognize that paralytic ileus is a common differential diagnosis in the early postoperative period. Although both paralytic ileus and SOO present with abdominal distension, nausea, and delayed bowel function, ileus usually arises immediately after surgery and tends to resolve spontaneously, whereas SOO often develops later and is localized to the stoma site. Several included studies have highlighted the need to distinguish between these two entities to avoid delayed recognition and treatment of SOO [5, 9, 20, 25]. While imaging may not always provide a definitive diagnosis, it plays a crucial role in excluding other potential causes of obstruction, such as intra-abdominal collections or leaks. Management of SOO closely parallels that of other benign bowel obstructions, beginning with conservative measures as long as the patient is clinically stable. Trans-stomal decompression offers a distinct advantage, as it provides surgeons with additional time before closing the stoma, allowing them to confirm the integrity of the distal anastomosis and rule out complications like leaks or strictures.

### Clinical significance & recommendations

Identifying and addressing risk factors significantly associated with SOO, such as RA muscle thickness, loop ileostomy, and HOS, is crucial to minimizing the serious consequences of this condition. This study highlights the clinical importance of RA muscle thickness, as increased thickness was found to substantially elevate the risk of SOO. In patients with this risk, surgeons may consider selecting a thinner and more lateral portion of the RA during stoma creation. Additionally, employing protective measures like increasing the aperture size can further reduce the risk. Similarly, the elevated risk of SOO linked to loop ileostomy also underscores the need to consider DEI, especially in patients with other predisposing factors. Patients with HOS require closer postoperative monitoring and careful fluid and electrolyte management to prevent dehydration and electrolyte imbalances that can exacerbate obstruction. It is equally important to rule out underlying causes, such as infections or anastomotic leaks, which could be the cause of the condition. These steps ensure that the patient’s overall health is maintained while addressing the mechanical complications associated with SOO.

Moreover, intraoperative measures can also significantly reduce the risk of SOO. Techniques such as stoma maturation using the OS approach, ensuring there is no twist at the mesentery, and avoiding angulation of the stoma limbs are effective strategies. Additionally, positioning the loop ileostomy slightly more proximally in cases of IPAA can help reduce tension in the stoma limb, thereby lowering the high risk of SOO in IPAA. Also, a multidisciplinary approach involving colorectal surgeons, dietitians, and stoma care nurses can ensure comprehensive care for high-risk patients. Recommendations include regular follow-ups with early imaging if obstruction symptoms arise and the establishment of guidelines for timely intervention. Further research is needed to validate these findings in more diverse populations and to explore other potential risk factors for SOO. By incorporating these clinical insights into routine practice, healthcare providers can significantly reduce the burden of SOO and improve the quality of life for patients requiring stoma creation.

### Limitations

Multiple limitations should be highlighted in this meta-analysis. First, the included studies were all retrospective in nature, which may introduce biases such as recall and selection bias, potentially affecting the reliability of the reported risk factors. Additionally, the majority of the included studies were conducted in Japan, limiting the generalizability of the findings to populations with different healthcare systems, surgical techniques, and patient characteristics. Furthermore, the relatively small number of studies and the focus on only four key variables further constrain the scope of the analysis, potentially overlooking other important risk factors for SOO. Lastly, although efforts were made to assess publication bias using funnel plots and the LFK index, the presence of minor asymmetry suggests the potential for underreporting of studies with negative or insignificant results, which could skew the meta-analysis. These limitations highlight the need for prospective, multicenter studies worldwide with standardized diagnostic criteria to validate and expand on the findings of this review.

## Conclusion

This systematic review and meta-analysis identified significant risk factors for stoma outlet obstruction, including increased rectus abdominis muscle thickness, high output stoma, and loop ileostomy. We also reported some other risk factors that were found to have significant association with SOO, including: ileal pouch–anal anastomosis, ileal pouch-to-ileostomy distance, oral inferior technique in constructing the stoma, smaller aperture size, higher BMI, and increased subcutaneous fat thickness at the stomal site. Effective management often relied on conservative approaches, while severe cases required surgical interventions such as stoma revision or early closure. The findings support several recommendations to mitigate the risk of SOO. Intraoperative measures, including stoma maturation using the oral superior technique, ensuring no twist at the mesentery, avoiding stoma limb angulation, and creating the stoma slightly more proximally in cases of ileal pouch-anal anastomosis to reduce limb tension, are highlighted as effective strategies. Additionally, optimizing aperture size during stoma construction was found to be a modifiable factor to balance the risks of SOO and parastomal hernia. Postoperatively, patients with HOS require close monitoring and fluid management to prevent dehydration and electrolyte imbalances. Collectively, these targeted strategies emphasize the need for tailored surgical techniques and vigilant postoperative care to reduce SOO incidence and improve patient outcomes.

## Supplementary Information

Below is the link to the electronic supplementary material.

Supplementary Material 1

## Data Availability

No datasets were generated or analysed during the current study.

## References

[1] Hanna MH, Vinci A, Pigazzi A (2015) Diverting ileostomy in colorectal surgery: when is it necessary? Langenbecks Arch Surg 400(2):145–152 25633276 10.1007/s00423-015-1275-1

[2] Santos FDCGG, Barbosa LER, de Araújo Teixeira JPM (2024) Ileostomy: early and late complications. J Coloproctology 44(1):E80–E86

[3] Maemoto R (2021) Risk factors and management of stoma-related obstruction after laparoscopic colorectal surgery with diverting ileostomy. Asian J Surg 44(8):1037–1042 33549406 10.1016/j.asjsur.2021.01.002

[4] Common terminology criteria for adverse events (CTCAE) (2017) Cancer therapy evaluation program (CTEP). Available from: https://ctep.cancer.gov/protocoldevelopment/electronic_applications/ctc.htm#ctc_50

[5] Fujii T et al (2015) Outlet obstruction of temporary loop diverting ileostomy. Hepatogastroenterology 62(139):602–605 26897937

[6] Oliveira L et al (1997) Laparoscopic creation of stomas. Surg Endosc 11(1):19–23 8994982 10.1007/s004649900287

[7] Okita Y et al (2017) Clinical characteristics of stoma-related obstruction after ileal pouch-anal anastomosis for ulcerative colitis. J Gastrointest Surg 21(3):554–559 27896653 10.1007/s11605-016-3329-2

[8] (2016) The ASCRS Textbook of Colon and Rectal Surgery TLH, Scott R Steele, Thomas E Read, Theodore J Saclarides, Anthony J Senagore, Charles B Whitlow, Editor. Springer Cham. p. 1292.

[9] Enomoto H et al (2021) Risk of outlet obstruction associated with defunctioning loop ileostomy in rectal cancer surgery. Cancer Diagn Progn 1(5):465–470 35403166 10.21873/cdp.10062 PMC8962870

[10] Fujii Y (2025) A novel technique for the construction of an end ileostomy to prevent stoma outlet obstruction after rectal resection and total colectomy: a single-center retrospective study. Surg Today. 10.1007/s00595-024-02956-1 39643755 10.1007/s00595-024-02956-1

[11] Ihara K et al (2024) Risk factors for stoma outlet obstruction after proctocolectomy for ulcerative colitis. J Anus Rectum Colon 8(1):18–23 38313747 10.23922/jarc.2023-018 PMC10831979

[12] Imaizumi Y (2024) High-output stoma is a risk factor for stoma outlet obstruction in defunctioning loop ileostomies after rectal cancer surgery. Surg Today 54(2):106–112 37222815 10.1007/s00595-023-02704-x

[13] Michonska I et al (2023) Nutritional issues faced by patients with intestinal stoma: a narrative review. J Clin Med 12(2) 10.3390/jcm12020510 PMC9862496 36675439

[14] Hara Y (2020) Organ/space infection is a common cause of high output stoma and outlet obstruction in diverting ileostomy. BMC Surg 20(1):83 32345295 10.1186/s12893-020-00734-7 PMC7189461

[15] Kumano K (2023) A comparative study of stoma-related complications from diverting loop ileostomy or colostomy after colorectal surgery. Langenbecks Arch Surg 408(1):139 37016188 10.1007/s00423-023-02877-6

[16] Caprino P (2023) Risk factors and outcomes of restorative proctocolectomy with ileal pouch-anal anastomosis for ulcerative colitis. Retrospective study of 75 single center cases. Eur Rev Med Pharmacol Sci 27(5):1945–1953 36930489 10.26355/eurrev_202303_31559

[17] Abe T et al (2021) Risk factors for outlet obstruction in patients with diverting ileostomy following rectal surgery. J Anus Rectum Colon 5(3):254–260 34395937 10.23922/jarc.2021-007 PMC8321594

[18] Sasaki S et al (2021) Risk factors for outlet obstruction after laparoscopic surgery and diverting ileostomy for rectal cancer. Surg Today 51(3):366–373 32754842 10.1007/s00595-020-02096-2

[19] Kuwahara K et al (2022) Risk factors for stoma outlet obstruction: preventing this complication after construction of diverting ileostomy during laparoscopic colorectal surgery. JMA J 5(2):207–215 35611234 10.31662/jmaj.2021-0187 PMC9090553

[20] Matsumoto Y (2024) Complications associated with loop ileostomy: analysis of risk factors. Tech Coloproctol 28(1):60 38801595 10.1007/s10151-024-02926-2

[21] Mori R et al (2022) Long distance between the superior mesenteric artery root and bottom of the external anal sphincter is a risk factor for stoma outlet obstruction after total proctocolectomy and Ileal-pouch anal anastomosis for ulcerative colitis. Ann Gastroenterol Surg 6(2):249–255 35261950 10.1002/ags3.12512 PMC8889852

[22] Tamura K et al (2019) Defunctioning loop ileostomy for rectal anastomoses: predictors of stoma outlet obstruction. Int J Colorectal Dis 34(6):1141–1145 31055627 10.1007/s00384-019-03308-z

[23] Kitahara T (2020) Risk factors for postoperative stoma outlet obstruction in ulcerative colitis. World J Gastrointest Surg 12(12):507–519 33437402 10.4240/wjgs.v12.i12.507 PMC7769745

[24] Okada S et al (2018) Elevated risk of stoma outlet obstruction following colorectal surgery in patients undergoing ileal pouch-anal anastomosis: a retrospective cohort study. Surg Today 48(12):1060–1067 30046881 10.1007/s00595-018-1698-8

[25] Ohira G (2018) Incidence and risk factor of outlet obstruction after construction of ileostomy. J Anus Rectum Colon 2(1):25–30 31583319 10.23922/jarc.2017-034 PMC6768823

[26] Dafnis G (2016) Early and late surgical outcomes of ileal pouch-anal anastomosis within a defined population in Sweden. Eur J Gastroenterol Hepatol 28(7):842–849 26945126 10.1097/MEG.0000000000000618

[27] Dolejs S, Kennedy G, Heise CP (2011) Small bowel obstruction following restorative proctocolectomy: affected by a laparoscopic approach? J Surg Res 170(2):202–208 21474147 10.1016/j.jss.2011.03.004 PMC3326606

[28] Francois Y (1989) Small intestinal obstruction complicating ileal pouch-anal anastomosis. Ann Surg 209(1):46–50 2535923 10.1097/00000658-198901000-00007 PMC1493886

[29] Kameyama H et al (2018) Small bowel obstruction after ileal pouch-anal anastomosis with a loop ileostomy in patients with ulcerative colitis. Ann Coloproctol 34(2):94–100 29742859 10.3393/ac.2017.06.14 PMC5951091

[30] MacLean AR (2002) Risk of small bowel obstruction after the ileal pouch-anal anastomosis. Ann Surg 235(2):200–206 11807359 10.1097/00000658-200202000-00007 PMC1422415

[31] Marcello PW (1993) Obstruction after ileal pouch-anal anastomosis: a preventable complication? Dis Colon Rectum 36(12):1105–1111 8253005 10.1007/BF02052257

[32] Mizushima T et al (2017) Risk factors of small bowel obstruction following total proctocolectomy and ileal pouch anal anastomosis with diverting loop-ileostomy for ulcerative colitis. Ann Gastroenterol Surg 1(2):122–128 29863130 10.1002/ags3.12017 PMC5881312

[33] Takehara Y (2022) A technique for constructing diverting loop ileostomy to prevent outlet obstruction after rectal resection and total colectomy: a retrospective single-center study. Surg Today 52(4):587–594 34689284 10.1007/s00595-021-02381-8 PMC8948144

